# Epstein-Barr Virus and Systemic Autoimmune Diseases

**DOI:** 10.3389/fimmu.2020.587380

**Published:** 2021-01-07

**Authors:** Gunnar Houen, Nicole Hartwig Trier

**Affiliations:** ^1^ Department of Biochemistry and Molecular Biology, University of Southern Denmark, Odense, Denmark; ^2^ Department of Neurology, Rigshospitalet, Glostrup, Denmark

**Keywords:** antibodies, Epstein-Barr virus, connective tissue disease, systemic autoimmune diseases, human herpes virus

## Abstract

Epstein-Barr Virus (EBV) is an extremely successful human herpes virus, which infects essentially all human beings at some time during their life span. EBV infection and the associated immune response results in production of antibodies (seroconversion), which occurs mainly during the first years of life, but may also happen during adolescence or later in life. Infection of adolescents can result in infectious mononucleosis, an acute serious condition characterized by massive lymphocytosis. Transmission of EBV mainly occurs through saliva but can rarely be spread through semen or blood, e.g. through organ transplantations and blood transfusions. EBV transmission through oral secretions results in infection of epithelial cells of the oropharynx. From the epithelial cells EBV can infect B cells, which are the major reservoir for the virus, but other cell types may also become infected. As a result, EBV can shuttle between different cell types, mainly B cells and epithelial cells. Moreover, since the virus can switch between a latent and a lytic life cycle, EBV has the ability to cause chronic relapsing/reactivating infections. Chronic or recurrent EBV infection of epithelial cells has been linked to systemic lupus erythematosus and Sjögren’s syndrome, whereas chronic/recurrent infection of B cells has been associated with rheumatoid arthritis, multiple sclerosis and other diseases. Accordingly, since EBV can shuttle between epithelial cells and B cells, the systemic autoimmune diseases often occur as overlapping syndromes with symptoms and characteristic autoantibodies (e.g. antinuclear antibodies and rheumatoid factors) reflecting epithelial and/or B cell infection.

## Introduction

### Epstein-Barr Virus

Epstein-Barr Virus (EBV) is a lymphotropic herpes virus and the causative agent of infectious mononucleosis (IM) (1–4). It was originally discovered in cells isolated from African Burkitt’s lymphoma and first later on, was it recognized that EBV is highly prevalent worldwide (5).

EBV is a member of the Human Herpes Viruses (HHVs) family, comprising eight viruses distributed on three subfamilies (Alpha, Beta, Gamma). EBV, which is also called HHV4, belongs to the Gammaherpesviridae, genus Lymphocryptovirus (6, 7). The circular double-stranded genome of EBV is approximately 172 kilobases, with more than hundred genes coding for approximately 85 proteins (**Table 1**) and approximately 50 non-coding RNAs (8–12).

**Table 1:** Epstein-Barr virus (EBV) proteins and their functions.

Function	Protein
*Entry glycoproteins*	BLLF1 (gP350), BZLF2 (gP42), BMRF2m, BXLF2 (gH), BKRF2 (gL), BALF4 (gP110), BLRF1 (gN), BHLF1, BDLF2
*Lytic replication*	BRRF1, BZLF1, BRLF1, BMRF1 (EA/D), BSLF1, BBLF4, BBLF2/3, BALF5, BALF2
*Viral DNA synthesis*	BORF2, BaRF1, BXLF1, BLLF3, BKRF3, BMLF1/BSLF2
*Late gene expression*	BGLF4, BGLF3, BcRF1, BFRF2, BDLF4, BVLF1, BDLF3.5 BFLF1, BFRF1A, BBRF1, BGRF1/BDRF1, BALF3, BGLF1, BVRF1
*Packaging and translocation of viral DNA*	
*Capsid*	BCLF1 (VCAp160), BFRF3 (VCAp18), BORF1, BDLF1, BVRF2, BdRF1 (VCAp40)
*Tegument*	BNRF1 (VCAp143), BPLF1, BSRF1, BBRF2, BGLF3.5, BGLF2, BTRF1, BLRF2 (VCAp23), BRRF2, BKRF4
*Virion assembly and egress*	BFLF2, BFRF1, BBRF3 (gM), BXRF1, BOLF1, BBLF1
*Latency* Stage I: Stage II: Stage III:	EBNA1 EBNA5, LMP1, LMP2A, LMP2B EBNA2, EBNA3, EBNA4, EBNA6
*Lytic immune-modulators*	BCRF1 (vIL-10), BARF1, LF2, BNLF2a, BDLF3 (gp150), BILF1, BHRF1 (EA/R), BALF1, BGLF5
*Uncharacterized proteins*	BLLF2, BNLF2b, BWRF1, LF3, LF1, RPMS1, A73, BARF0, BILF2

Several strains of EBV exist. The first EBV variants identified were type 1 (type A) and type 2 (type B). While type 1 (B95-8, GD1, and Akata) is the main EBV type prevalent worldwide, type 2 (AG876 and P3HR-1) is as abundant as type 1 in sub-Saharan Africa (13). The EBV variants have different replicative properties and individuals may become superinfected with two or more strains (14–16).

**Figure 1:**
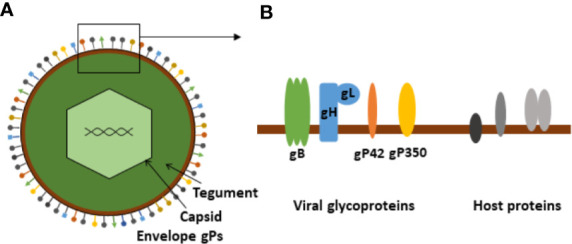
Schematic presentation of Epstein-Barr virus. **(A)** Schematic illustration of the basic EBV structure. **(B)** Enlargement of membrane section showing viral envelope glycoproteins (entry complex) and putative host-derived membrane proteins.

The structure of EBV is typical of HHVs and related viruses (**Figure 1**) (17). It has an outer lipid envelope, derived from the producing host cell, wherein several viral proteins are embedded in addition to host cell-derived membrane proteins. Many of the viral envelope membrane proteins are glycoproteins (gPs). Currently, 13 gPs have been identified, 12 of which are expressed only during the productive, lytic replication cycle and one of which (BARF1, a decoy viral colony-stimulating factor 1 receptor (vCSF1R)) may be expressed during latency as well. Some of these are listed in **Table 1** (18). Inside the envelope is the viral tegument, in which the capsid is embedded with its enclosed genome and associated proteins.

The life cycle of EBV is characteristic of a large enveloped DNA virus, being composed of primary infection, latency, and lytic reactivation phases. In addition, EBV has an ability to infect several cell types (19). The EBV genome encodes 9 different envelope entry gPs (**Table 1**). The functions of all of these are not completely understood, but the roles of the most important gPs are known in much detail. The tropism of newly released EBV virions is determined by the envelope gPs, which in turn vary somewhat depending on the host cell (20). The major cell types infected by EBV are epithelial cells and B cells. Epithelial cells are the first cell type to be infected, as EBV is transmitted to recipients through saliva. Next, B cells are infected when EBV gains access to the underlying tissue after release from the oropharyngeal epithelium (21–25). EBV virions released from epithelial cells have a preference for B cells and EBV virions released from B cells have a preference for epithelial cells, due to the composition of the envelope gPs (20, 26, 27).

Epithelial cell infection may occur by direct fusion of the viral envelope membrane with the plasma membrane of the target cell. Attachment of the virus to the cell surface primarily occurs *via* gH/gL interaction with Ephrin A2 (EphA2) and αvβ5/αvβ6/αvβ8 integrins and *via* BMRF1, which interacts with β1 integrins, but EBV gP350/220, which interact with complement receptor (CR)2 (CD21) and CR1 (CD35) also plays a role in epithelial cell attachment. The gH/gL interaction with integrins is mediated by a KGD motif on gH, and the interaction between gH/gL and EphA2 occurs through the receptor’s ligand binding and fibronectin type III repeats and is mediated by the gP42 binding site on gH. Upon attachment and interaction with integrins or EphA2, a conformational change in gH/gL allows interaction with the trimeric gB, which in turn changes conformation and facilitates viral entry by acting as a fusogen (20, 28–32).

Other EBV proteins may also play a role during infection of epithelial cells, e.g. BMRF2, which can bind integrin αvβ1 and BDLF2, which can bind non-muscle myosin heavy chain IIA. Moreover, gB, itself can bind neuropilin-1 and IgA directed to EBV envelope proteins may enhance infection through the polymeric IgA receptor (28, 32–36).

B cell infection is mediated by gP350/220, which binds CR2 and CR1, together with gP42, leading to the formation of a complex together with major histocompability complex (MHC)-II (37). Upon attachment, the virion is endocytosed and gH/gL can form a fusion complex with gP42-MHC-II, thus inducing a conformational change in gH/gL (similar to what happens upon gH/gL interaction with integrins and/or EphA2). As a result, trimeric gB changes conformation and promotes fusion of the viral membrane with the endosome membrane, thus releasing the virus to the cytoplasm (**Figures 1** **–** **3**) (20, 26, 27). The structural and mechanistic basis of B cell entry has been elucidated in much detail by solving the structures of gB, gP42, complexes of gH/gL, gP42/MHC-II (human leukocyte antigen (HLA)-DR1) and of gH/gL/gP42/MHC-II in pre- and post-fusion conformations (20, 38, 39). This has allowed modelling not only of the EBV B cell entry complex, with the involved gPs acting sequentially and in concert, but also of the epithelial cell entry complex. Thus, gH/gL/gB appears to constitute a core entry machinery and gP42 seems to be a primary determinant of EBV tropism, since it participates in and promotes B cell infection but inhibits epithelial cell infection by binding to the EphA2/integrin-binding site(s) on gH/gL (20).

**Figure 2:**
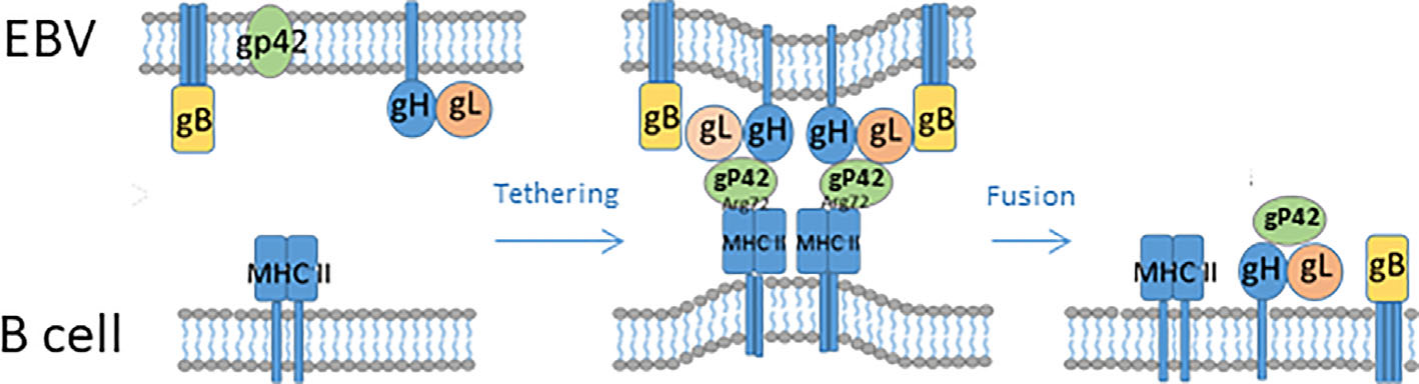
Schematic illustration of EBV fusion with the cellular lipid bilayer of B cells. For gP42 to become active, the protein is cleaved N-terminally. gP42 interacts with gH/gL, and the complex interacts with gB. gP42 interacts with the β1 domain of MHC-II, which ultimately results in membrane fusion.

**Figure 3:**
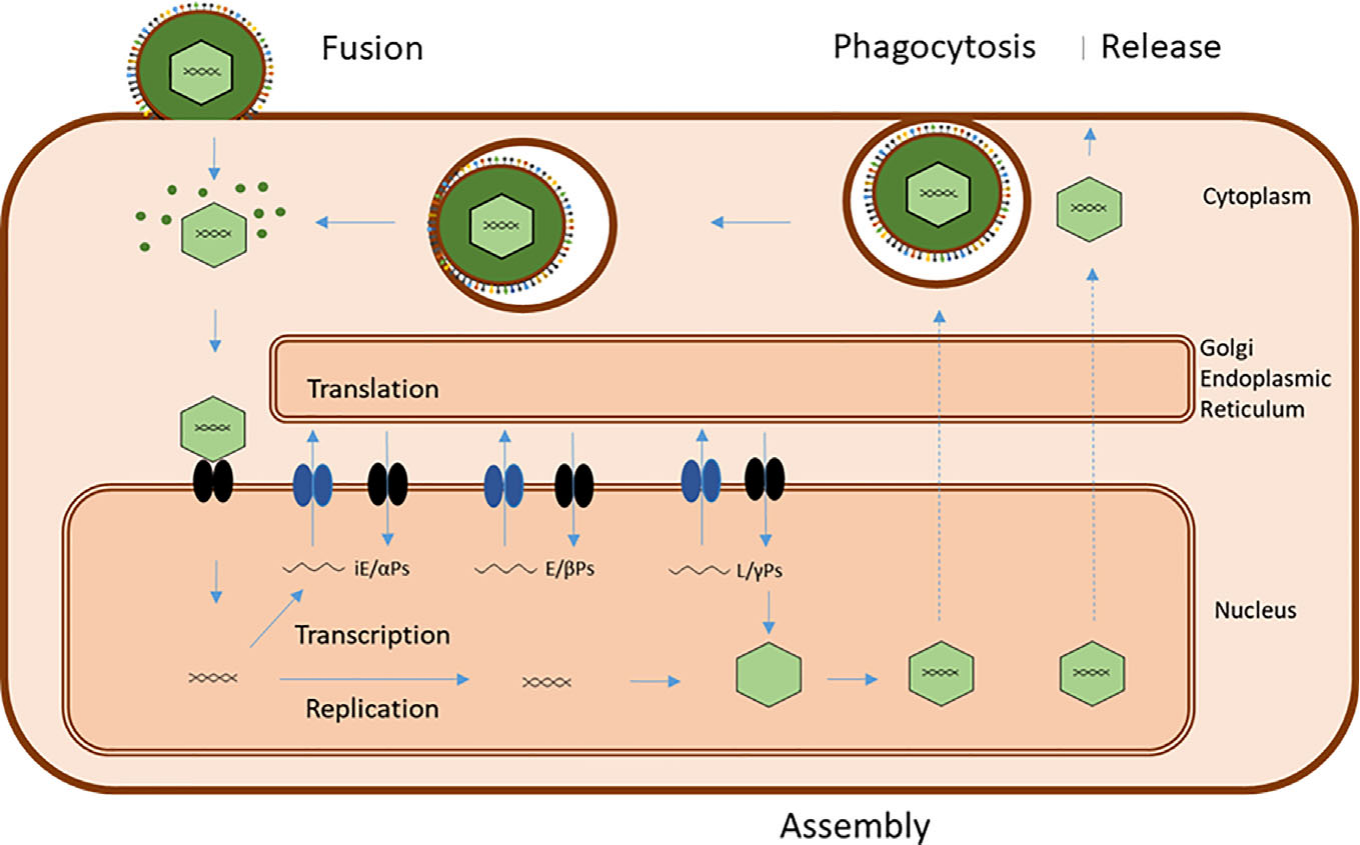
Common basic EBV infection scheme. Viral entry can occur by direct fusion of the viral plasma membrane-derived envelope with the target cell membrane or by endocytosis/phagocytosis of virus followed by fusion of virus envelope and endosome/phagosome membrane. Both processes release virions and viral tegument proteins into the cytoplasm. Released virions are transported to the nuclear membrane and the viral genome introduced into the nucleus together with associated proteins. This initiates transcription of viral genes in a sequence of immediate-early (iE) genes, coding for regulatory alfa-proteins, early genes, coding for catalytic beta-proteins, and late (L) genes, coding for structural gamma proteins. Translation of viral messenger RNAs takes place on ribosomes in the cytoplasm and on the endoplasmic reticulum, and the viral proteins are routed to different locations for subsequent virus assembly. Successful replication of viral genomes and transport of capsid proteins to the nucleus results in assembly of virions, which travel to the plasma membrane by a series of envelopment/fusion events involving intracellular membranes (stippled lines) ending with budding of mature virus with a plasma membrane envelope, containing viral glycoproteins and host-derived membrane proteins. Premature cell death releases a mixture of “naked” virions and diffentially enveloped viruses.

Successful entry and viral take-over of cellular control leads to an ordered sequence of transcription of viral genes, translation of viral mRNAs and finally, replication of the viral DNA and assembly of new virus (**Figure 3**). The virion assembly and egress from the host cell utilizes the host cell exocytosis machinery and involves several viral proteins apart from the structural, tegument and envelope proteins (**Table 1**) but is less understood than the entry process (40, 41). Collectively, EBV utilizes several characteristic major host cell membrane proteins for entry and release and due to the properties of gP42, it has a preference for epithelial cell infection when produced by B cells and vice versa, assuring that some virions will eventually return to salivary gland cells and be able to be transmitted to new individuals.

Infection activates the intracellular antiviral mechanisms and induces an extracellular immune response against EBV antigens, with generation of specific helper T cells, antibodies and cytotoxic T cells and activation of natural killer (NK) and NK T cells (NKT) (42–46). In response to this, EBV has evolved mechanisms for evading the extracellular innate immune system and the host cell’s innate antiviral systems together with adaptive immune system evasion mechanisms and the virus devotes a substantial part of its proteins and non-coding RNAs to this (47–51). Together, the innate and adaptive immune evasion mechanisms of EBV assure its persistence in the host. A major aspect of the immune evasion strategy is EBV’s ability to enter a latent state with minimal expression of viral genes and minimal presentation of viral peptides to the immune system (19, 52–54). This mainly occurs in (memory) B cells, but latency may also take place in epithelial cells. From the latent state, EBV can occasionally reactivate, e.g. in response to antigen stimulation of memory B cells, resulting in lytic production of virions upon expression of an ordered sequence of viral genes (55–57). This in turn mounts an increased immune response against EBV, neutralizing infected cells and forcing the virus into latency again. Reactivation may also occur upon “waning” of the cellular immunity to the virus and infected individuals through the rest of their lives experience a persistent “battle” with EBV. Depending on the host immune system and environmental factors, some individuals may eventually suffer from EBV-related diseases, either as a result of EBV immune evasion or as a result of EBV infection of other cell types (T cells, NK cells, NKT cells, monocytes/macrophages, and others), which may take place in some instances.

### Epstein-Barr Virus Immune Evasion

As a part of the common evolutionary history of humans and EBV, the virus has evolved a multitude of immune evasion mechanisms, including wrapping itself in host cell-derived membranes (envelopment) and the ability to switch between latent and lytic life stages (50, 58). Most of the immune evasion proteins of EBV are expressed during the lytic cycle and some are shown in **Table 1** as “immune modulators”. More EBV proteins are presumably involved in immune evasion and many EBV proteins serve two or more functions.

In the latent state, as mentioned above, there is minimal expression of viral genes and minimal presentation of viral peptides to the immune system (19, 52–54). In the “deep” latency state, only EBV nuclear antigen (EBNA)1, which assures maintenance and replication of the EBV genome along with host cell chromosomes, is expressed. In order to avoid presentation of EBNA1-derived peptides on MHC-I, the EBNA1 protein contains a characteristic AG repeat sequence, which interferes with proteasome processing and which interacts with nucleolin to restrain its expression. Moreover, EBNA1 also contains characteristic RG repeat sequences, which may play a role in immune evasion (59–61). Upon switching to lytic cycle with production of viral proteins, EBV downregulates MHC-I and interferes with presentation of viral peptides on MHC-I *via* BDLF3-induced ubiquitination of MHC-I (62). Likewise, in B cells, EBV can also downregulate MHC-II by BDLF3-induced ubiquitination of MHC-II (62) and gP42 can be released in a soluble form, which inhibits interaction between MHC-II and the T cell receptor (63, 64). Other EBV proteins are involved in minimization of MHC-I expression, including BNLF2a, BILF1, BGLF5. The exonuclease BGLF5 degrades cellular mRNAs including those for MHC-I and BILF1 associates with cell surface MHC-I and enhances its degradation, while BNLF2a prevents MHC-I peptide loading by inhibiting the transporter associated with peptide loading (TAP) (65–68). As a means to avoid NK cell recognition, EBV upregulates non-classical MHC during the phase of viral protein synthesis. Lytic production of viral proteins and RNAs as well as replication of viral DNA requires that EBV can prevent cellular apoptosis and EBV has evolved an elaborate set of proteins for pacifying intracellular virus-sensing apoptosis-inducing mechanisms including downregulation and inhibition of toll-like receptors (47, 49, 50, 62, 68–71).

EBV also produces soluble mediators, which interfere with mobilization of the adaptive immune system. BCRF1 encodes a viral IL10 homologue (vIL10), which dampens inflammation (72–75) and, as mentioned above, BARF1, encodes a decoy vCSF1R, which binds CSF1 and thereby limits mobilization of hemopeoietic stem cells (76, 77).

The viral envelope derived from the host cell (**Figure 2**) offers substantial protection to the enclosed viral particle by mimicking a host extracellular vesicle. In principle, the viral envelope may contain all host-derived membrane proteins relevant for “disguise” and immune evasion (e.g. MHC molecules, complement regulators, Fc receptors, phagocytosis-inhibitory (“don’t-eat-me”) molecules, etc.). However, to be able to exit from the host cell in a controlled process, and to be able to infect other cells, several viral gPs have to be inserted into the envelope membrane as mentioned above. These proteins are targets for innate immune recognition and antibody (Ab) production, as described in the preceding paragraph, but extensive glycosylation with host-derived glycans affords considerable protection against pattern recognition (scavenger) receptor (including complement) and Ab recognition (“glycan shielding”). Moreover, as described, some of the immune reactions may actually be exploited for viral infection and spreading, f.ex. “hitchhiking” with complement/CRs (e.g. EBV entry in B cells) or with Abs bound to viral envelope gPs/Fc receptors (FcRs) (e.g. cytomegalovirus entry in monocytes/macrophages or EBV entry in B cells with cell surface immunoglobulins (Igs) against EBV envelope gPs) (78–80). Despite the many immune evasion mechanisms of EBV, the normal healthy human immune system is able to eradicate active virus and force it into a quiescent (“immune silent”) state (latency). Since EBV appears to be able to evade most or all innate immune system components, the final “victory” of the immune system must rely on cellular immune control of EBV involving a combination of T cells, NK cells and NKT cells, in accordance with all available evidence of EBV immunity. The molecular details of how this results in EBV latency instead of cell killing are not known, but it is firmly established that EBV has evolved mechanisms of latency as an ultimate, opportunistic and effective immune evasion strategy.

### Epstein-Barr Virus Epidemiology

A majority of children becomes infected with EBV early in life and seroconversion, the appearance of Abs to EBV peaks around 1–2 years of life, where the majority of infectious cases is non-complicated and may even go unnoticed. A second peak in seroconversion is seen in puberty, due to increased frequency of close social contact with already infected persons. Infection in adolescence is more problematic and may result in IM in many cases, popularly denoted “kissing disease” (1–4). For the majority of infected individuals latent infection does not appear to influence the general health, however, dysregulation of latency or inability to control the lytic infection may lead to development of lymphoproliferative diseases and lymphoma (81).

The course of EBV infection is determined by the virus load and an individuals’ immune system state, which in turn is determined by the person’s gene composition, other infection history and several environmental factors, which all may influence the immune capacity of a person to various degrees.

Genetic factors influencing EBV control are in principle all genes of the immune system. In practice, T cells, NK cells and NKT cells have turned out to be of utmost importance (42–44, 46). Relatively few studies have addressed genetic factors associated with EBV infection, presumably due to the ubiquitous occurrence of EBV. Consequently, since essentially all persons eventually become infected, genetic associations will only relate to the age of infection. Epidemiological studies have indicated an association of some MHC-II and -I alleles and EBV seropositivity. Moreover, mannan-binding lectin insufficiency has been linked to EBV seropositivity as well (82). Also, some polymorphisms in the (IL) 10 gene and other immune system genes have been linked with EBV seropositivity (83). However, all these studies are hampered by a relative scarcity of seronegative persons.

Besides from genetic factors, environmental factors are known to affect a person’s EBV status. Currently identified factors are sunlight/Vitamin D (VitD), smoking and body mass index (BMI) (84, 85). These factors may be assumed to influence the general immune status of individuals and thereby affect susceptibility to EBV infection. E.g. sunlight/VitD has been proposed to protect against autoimmunity by increasing the number of CD8+ T cells available to control EBV infection (84). Moreover, obesity has been proposed to impact the cellular immune response to infections and induce a state of chronic immune-mediated inflammation (85), but more studies are required to understand these associations. Finally, prior infections may play a role in shaping an individual’s immune repertoire and resulting capacity to combat later infections, as evidenced by the more serious course of EBV infection in adolescence or later in life.

### Epstein-Barr Virus Serology – Assays, Antigens

The presence of EBV nucleic acid material in infected persons can be determined by numerous methods, e.g. by direct sequencing, fluorescence in-situ hybridization (FISH) and polymerase chain reaction (PCR) analysis of blood samples for EBV-derived DNA or RNA, while (prior) infection/reactivation may also be demonstrated by PCR analysis of saliva (86–91). In relation to testing of EBV in biopsy tissues, molecular detection of EBV-encoded RNA transcripts by FISH remains the gold standard. Moreover, EBV-encoded RNA hybridization and EBV LMP1 immunostains are used routinely to detect latent EBV in tissues affected by posttransplant lymphoproliferative disorder (PTLD) or in enlarged nodes from IM patients (92). Traditionally, serology is the simplest way to test for EBV infection and even for evaluating acute versus remote infection in healthy individuals. High serological titers serve as a tumor marker for some EBV-related malignancies, but titers are not a dependable tumor marker in immunocompromised hosts. EBV viral load testing by quantitative DNA amplification of blood samples has proven useful for early diagnosis and monitoring patients with PTLD (92).

Acute infection may also be inferred from analysis of IgM to viral antigens, while prior infection may be inferred from the presence of IgG to EBV antigens, and IgA can be used as a measure of epithelial infection load (45). Using three EBV antigens, viral capsid antigen (VCA) IgG, VCA IgM and EBNA1 IgG, it is normally possible to distinguish an acute from a past infection. While the presence of VCA IgM and VCA IgG without EBNA-1 IgG indicates a current acute infection, does the presence of VCA IgG and EBNA1 IgG without VCA IgM typically indicate a past infection (93).

Among the 85 proteins encoded in the EBV genome, several have been used for detection of Abs to EBV including EBNA1, EBNA2, VCAp23, VCAp18, early antigen diffuse (EAD), gP350, BARF1 (**Table 1**) (15, 94–97). IM has previously been associated with the presence of so-called heterophile Abs, however, this test has a rather low specificity and it remains unclear, what the test actually measures (2, 98).

Since induction of Abs follows a pattern of viral Ag production, seropositivity will depend on a person’s ability to control EBV and the balance between latent and lytic EBV infection. Moreover, any assay has a characteristic sensitivity and specificity for EBV detection, and some individuals may be judged false negative or positive. Thus, to fully define the incidence and prevalence of EBV infection in a population, several assays should be used, preferably combining assays for detection of viral nucleic acids, Abs to different viral antigens and the frequency of virus-specific T cells. Optimally, different detection principles may also be used; e.g. for Ab detection: enzyme-linked immunosorbent assay (ELISA) and immunoblotting, for T cell detection: antigen-induced cytokine release and peptide-MHC tetramer assays, and the assays should target different parts of the viral genome or different viral antigens representing both latent and lytic states. This is evidently very labor-intensive but may be realized by using multiplex techniques.

### Epstein-Barr Virus and Diseases

Many diseases are known to be associated with EBV infection and prior IM increases the risk of many of these diseases (2, 99). IM itself is a prolonged state of fever, swollen lymph nodes, fatigue, malaise and various other symptoms. Few studies have focused on genetic factors associated with IM. Similar to EBV infection itself, some MHC-I and -II alleles and polymorphisms in the IL10 gene have been associated with IM development (82).

In contrast to the scarcity of information about genetic factors involved in EBV infection itself, several data has been published relating to EBV involvement in diseases and genetic factors associated with these. Several types of cancer, notably B cell lymphomas and nasopharyngeal epithelial carcinomas, affecting the two primary cell types targeted by the virus, are caused by EBV (99–102). This can be ascribed to EBV’s ability to evade cellular antiviral mechanisms and control cellular apoptotic pathways and to its capacity for immune evasion (103). However, several other diseases affecting other cell types, which may become infected by EBV are known, including T cell lymphomas, NK cell leukemias and other T cells, NKT cells and NK cell lymphoproliferative diseases (101, 104, 105). Moreover, several systemic autoimmune diseases (SADs) and multiple sclerosis (MS) have been demonstrated to be associated with chronically relapsing EBV infection and inefficient immune control of the virus.

### Systemic Autoimmune Diseases

SADs are a group of partly overlapping syndromes, also called connective tissue diseases, since they often are accompanied by inflammation of connective tissues. The SADs include the relatively common rheumatoid arthritis (RA) and the more rare conditions Sjögren’s syndrome (SS), systemic lupus erythematosus (SLE), systemic scleroderma (SSc), and others (**Table 2**) (106, 107).

**Table 2:** Systemic autoimmune diseases (SADs) and their characteristics.

Disease	Genetics	Environmental factors
Mixed connective tissue disease (MCTD)	HLA-DRB1, multiple genes	VitD, smoking, EBV, sunburn, silica dust
Polymyositis – dermatomyositis (PM-DM)	HLA-DRB1, multiple genes	Smoking
Rheumatoid arthritis (RA)	HLA-DRB1, PTPN22, multiple genes	VitD, smoking, EBV
Sjögren’s syndrome (SS)	HLA-DRB1, PTPN22, multiple genes	VitD, EBV, inverse correlation with smoking
Systemic lupus erythematosus (SLE)	HLA-DRB1, C’, multiple genes	VitD, smoking, EBV, sunburn, silica dust
Systemic sclerosis (SSc) HLA-DRB1, multiple genes Silica dust, solvents		

### Epstein-Barr Virus and Rheumatoid Arthritis

The clinical characteristics of RA are swollen and painful joints, caused by synovial inflammation eventually resulting in exaggerated connective tissue deposition (pannus formation) and bone erosion, with resulting disability. Moreover, RA is frequently accompanied by systemic complications such as vascular disease, osteoporosis, and others (108–110). Most RA patients have characteristic autoantibodies (AuAbs) including rheumatoid factors (RFs) and anti-citrullinated protein antibodies (ACPA)s, but many also have anti-nuclear Abs (ANAs) (111, 112). The etiology of RA is commonly ascribed to genetically determined defective self-tolerance, but environmental factors are known to play a dominating role, including EBV infection (113–116). Alleles of many genes are known to contribute to RA, notable HLA-DRB1 alleles containing shared epitope (SE) motives, but many other genes affecting the immune system and in particular lymphocytes have an impact (108, 110, 117). Tumor necrosis factor (TNF) plays an important role in a large proportion, if not most RA patients, and therapeutic Abs targeting TNF have good therapeutic efficacy in many patients (109, 118).

EBV evidently plays an important role in the etiology of RA, although not all evidence indicates an association between RA and EBV (119). Mechanisms behind the role of EBV in RA may include either molecular mimicry in the initiation of RA, bystander activation effects or chronic recurrent infection of joint epithelial cells and synovial B cells. The characteristic ACPAs seen in a major proportion of RA patients have been found to represent Abs to a citrullinated region of EBNA2, an important transcription factor of EBV expressed in lytic phases (120). Presumably, EBNA2 and possibly also other EBV proteins become citrullinated by peptidyl arginine deiminase (PAD) enzymes during the inflammatory process in RA joints (121, 122). RFs have been found to target cryptic epitopes of IgG heavy chains, presumably being released by lysis of EBV-infected B cells (123) and MHC-II molecules with SE motives (certain HLA-DRB1 alleles) have been found to be optimal ligands for EBV gP42, thus favoring EBV infection of B cells with these forms of MHC-II (31). Thus, the major characteristics of RA can be related to chronic EBV infection, and actually, serum EBV DNA has been found to correlate with disease activity (124). Furthermore, EBV has been demonstrated to be present in the synovium of RA patients (115, 125, 126).

### EBV and Sjögren’s Syndrome

SS is a disease resulting in progressive destruction of exocrine salivary and lacrimal gland tissue. The major clinical characteristics are xerostomia and xeropthalmia in addition to fatigue and various other symptoms, which may also affect other organ systems (127, 128). Patients most often have ANAs and characteristic AuAbs are Ro60 and La Abs, but various other AuAbs may also be present. In addition, RFs are present in a majority of patients, whereas ACPAs are usually absent (128).

The etiology of SS has been suggested to involve several environmental and genetic factors, molecular mimicry and bystander activation (129, 130). Genetic factors include certain MHC-II (especially some HLA-DRB1) alleles, some MHC-I alleles and components of the interferon regulatory system (131). Environmental factors include vitD deficiency, smoking, silica dust exposure and virus infections (129). Especially EBV infection has been associated with SS (132, 133). The mechanisms involved in SS are presumably similar to RA and other SADs, but are much less studied. RA and SS often co-exist and SS primarily affects the epithelial tissues targeted by EBV, i.e. salivary and lacrimal glands, making the association with EBV infection particularly attractive.

### Epstein-Barr Virus and Systemic Lupus Erythematosus

SLE is a disease, which clinically presents with a heterogenous array of symptoms, often evaluated by the SLE disease activity index (SLEDAI) or similar indexes, including complementemia, DNA Abs, leukopenia, thrombocytemia, fever, fatigue, skin rash, UV sensitivity, mucosal ulcers, alopecia, pleuriris or pericarditis, proteinuria, hematuria, nephritis, myositis, arthritis, vasculitis, headache, stroke, and more rarely, neuropsychiatric symptoms (134–137). The disease may show a relapsing/remitting course, depending on the efficacy of treatments (138, 139).

SLE has been described as an immune complex disease, since it is often associated with decreased levels of complement components (140). Other characteristics are the presence of ANAs, notably DNA Abs, which are included in the SLEDAI, but in many cases AuAbs to a heterogenous panel of AuAgs are present and changes in the AuAb profile may reflect changes in disease activity (141–144).

Genetically predisposing factors are first of all certain HLA-DRB1 alleles, but multiple immune system genes, including other MHC-II alleles and some MHC-I alleles, as well as genes affecting cellular waste removal, have been found to influence disease development (145, 146).

Major environmental factors promoting development of SLE are silica dust exposure, sun burn, smoking, vitD deficiency and EBV infection (147–153). The etiology has been suggested to involve molecular mimicry between EBV EBNA1 and cellular Ro 60, and/or bystander activation (154, 155).

Decreased immune control of chronic EBV infection has been found to be a contributing factor, if not a major cause (152, 156, 157), but other infections may also play a role in SLE development or exacerbation (158, 159). The presence of DNA Abs and other ANAs would seem to be compatible with infection by a DNA virus in combination with inefficient removal of apoptotic and necrotic material.

## Discussion

SADs constitute a group of partly overlapping autoimmune disease syndrome and include systemic sclerosis (SSc), mixed connective tissue disease (MCTD) and polymyositis/dermatomyositis (PM/DM) in addition to RA, SS, and SLE (**Table 2**). These diseases share several genetic and environmental factors, in particular the predisposing effect of certain HLA-DRB1 alleles (although not exactly identical alleles), the predisposing effect of EBV infection and of factors, which can be related to EBV infection (e.g. vitD deficiency) (**Table 2**) (106, 107, 160–171).

**Figure 4:**
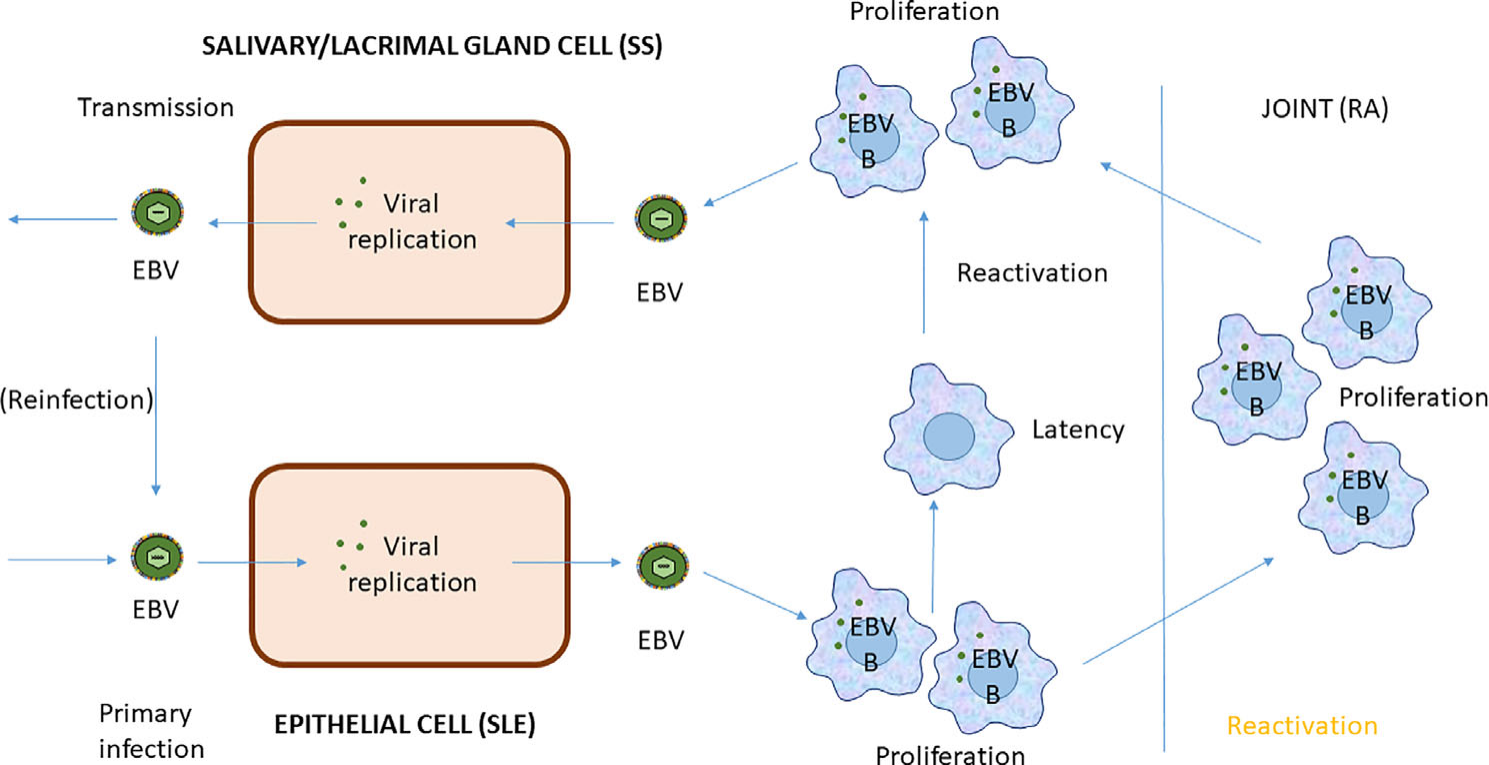
EBV Infection cycle in B cells of systemic autoimmune diseases.

The evidence for a major etiological role of EBV is particularly strong for RA, where several of the clinical characteristics can be related to EBV as described above (RFs, ACPAs, SE-allele disposition). Current treatments can also be related to EBV infection, e.g. CD20 monoclonal antibodies (MAbs), which presumably diminish the burden of EBV-infected (memory) B cells, and TNF MAbs, which possibly diminish the burden of EBV infection by an anti-inflammatory effect (172–174). The evidence for an etiological role of EBV in SLE is also strong and seems to point to EBV infection of epithelial cells in combination with decreased removal of apoptotic/necrotic cell debris (175). Thus, these two prototype SADs can be seen as the results of a chronic, poorly controlled, relapsing/remitting EBV infection targeting the two major host cells of EBV; B cells in RA and epithelial cells in SLE. In RA, relapses most likely follow re-activation of EBV in (memory) B cells upon Ag stimulation. This results in production of EBV-transformed B cell blasts, which by their very nature will attempt homing to bones and therefore will have a tendency to populate joints, where the concomitant lytic EBV production may also result in EBV infection of synovial epithelial cells. In SLE, B cells will also be involved, thus accounting for the common involvement of joints and other symptoms overlapping with RA, however, the major target cells affected are epithelial cells, thus accounting for the common skin and mucosal pathology, while the defective removal of EBV and cellular debris results in immune complex deposition in affected organs and in particular kidneys, by virtue of their filtrating actions. SS has been studies less intensively than RA and SLE but the relation to EBV is nevertheless even more obvious. In SS, pathological symptoms reminiscent of both RA and SLE are seen. This again reflects the tendency of EBV to “shuttle” between B cells and epithelial cells and in particular the ability of EBV to return to salivary (and lacrimal) gland epithelial cells as part of its natural life cycle (**Figure 4**). Thus, SS may in some respects be thought of as SLE effecting the exocrine glands, while SS also has many characteristics in common with RA.

Other autoimmune diseases, especially MS have also been found to depend on EBV infection in several aspects (176). The question therefore arises, how EBV can be involved in these apparently diverse diseases? A common feature seems to be decreased immune control of EBV. T cells are crucial for the control of EBV (and other viruses) and defective/exhausted T cell repertoires are characteristic of SADs (177). This allows for chronic infections with continuous cycles of relapses and remissions. However, while this may explain a common involvement of EBV (or other viruses) in disease etiology, it does not explain the different clinical appearances and the differences in e.g. association with different HLA alleles. A plausible explanation is that the role of EBV does not depend solely on e.g. entry, which in RA seems to be facilitated by SE-containing HLA alleles. Other HLA interactions must also be involved, e.g. presentation of EBV and/or host peptides, interactions with the peptide loading complex, interaction with other EBV or host proteins, etc. In general will the genetic composition of the host determine the fate of EBV in different cell types, including the interactions of EBV attachment and entry proteins with the target cell membrane proteins, the ability of the host cell to undergo apoptosis and the possibility to support lytic production of virus, and the efficiency of adaptive immune control of EBV. Since there are large differences in individual immune systems and in infection histories, one possibility for the different appearances of EBV-related diseases could also be individual mutations in EBV genomes during chronic infections and/or re-infections, and/or different rates of co-infection with other viruses. Patients with SADs are often prone to various infections, possibly due to inherent or acquired immune deficiencies, which predispose to coinfection with other viruses e.g. cytomegalovirus and others, which have been suggested to play a role in SAD development (178–181).

Patients with SADs also have increased tendency to develop cancer, including various forms of lymphoma. This may relate to secondary effects of treatment with immuno-suppressive drugs but may also reflect an inherent ability of EBV to cause transformation of B cells and epithelial cells (13, 99–102, 160).

## Conclusion

EBV has been found to play a role in several, if not all SADs. It remains unclear, whether the role of EBV is primarily in initiation of disease (e.g. by molecular mimicry) or is simply due to the chronic relapsing-remitting nature of EBV infections. Many characteristics of especially RA can be ascribed to EBV infection, but this may also be the case for other SADs. Future studies should focus on interaction of EBV proteins and non-coding RNAs with host molecules and on the role of other viruses in relation to EBV infection.

## Author Contributions

All authors contributed to the article and approved the submitted version.

## Conflict of Interest

The authors declare that the research was conducted in the absence of any commercial or financial relationships that could be construed as a potential conflict of interest.
